# Comparative Immune- and Stress-Related Transcript Response Induced by Air Exposure and *Vibrio anguillarum* Bacterin in Rainbow Trout (*Oncorhynchus mykiss*) and Gilthead Seabream (*Sparus aurata*) Mucosal Surfaces

**DOI:** 10.3389/fimmu.2018.00856

**Published:** 2018-05-02

**Authors:** Ali Reza Khansari, Joan Carles Balasch, Eva Vallejos-Vidal, David Parra, Felipe E. Reyes-López, Lluís Tort

**Affiliations:** Department of Cell Biology, Physiology and Immunology, Universitat Autònoma de Barcelona, Bellaterra, Spain

**Keywords:** mucosal immunity, cortisol, gene expression, skin, gills, gut

## Abstract

Fish have to face various environmental challenges that may compromise the efficacy of the immune response in mucosal surfaces. Since the effect of acute stress on mucosal barriers in fish has still not been fully elucidated, we aimed to compare the short-term mucosal stress and immune transcriptomic responses in a freshwater (rainbow trout, *Oncorhynchus mykiss*) and a marine fish (gilthead seabream, *Sparus aurata*) to bacterial immersion (*Vibrio anguillarum* bacterin vaccine) and air exposure stress in skin, gills, and intestine. Air exposure and combined (vaccine + air) stressors exposure were found to be inducers of the cortisol secretion in plasma and skin mucus on both species in a time-dependent manner, while *V. anguillarum* bacterin exposure induced cortisol release in trout skin mucus only. This was coincident with a marked differential increase in transcriptomic patterns of stress- and immune-related gene expression profiles. Particularly in seabream skin, the expression of cytokines was markedly enhanced, whereas in gills the response was mainly suppressed. In rainbow trout gut, both air exposure and vaccine stimulated the transcriptomic response, whereas in seabream, stress and immune responses were mainly induced by air exposure. Therefore, our comparative survey on the transcriptomic mucosal responses demonstrates that skin and gut were generally more reactive in both species. However, the upregulation of immune transcripts was more pronounced in gills and gut of vaccinated trout, whereas seabream appeared to be more stress-prone and less responsive to *V. anguillarum* bacterin in gills and gut. When fish were subjected to both treatments no definite pattern was observed. Overall, the results indicate that (1) the immune response was not homogeneous among mucosae (2), it was greatly influenced by the specific traits of each stressor in each surface and (3) was highly species-specific, probably as a result of the adaptive life story of each species to the microbial load and environmental characteristics of their respective natural habitats.

## Introduction

Fish are living in a microbial-loaded environment involving an intense interaction of their mucosal surfaces with microbiota and therefore various immune responses in these surfaces. The diversity of the potential environmental or imposed stressors (i.e., changes in temperature, photoperiod, pH, oxygen saturation, population density, pathogen load, and virulence) biases the efficacy and time course of the mucosal immune responses in a species-specific manner (1–4). Thus, defensive responses in fish show great interspecific diversity and agglutinate the mucosal-associated structures in a common mucosal immunity framework (5, 6). When interacting with mucosal interfaces, exogenous bacteria and viruses skew the immune responsiveness depending on each surface. Pathogens, such as *Vibrio anguillarum*, are able to adhere preferentially to fish integument (7), modifying the thickness, quality, and secretory pattern of skin immune defenses which, in turn, vary depending on the interspecific susceptibility to diseases, pathogen virulence, and environmental toxicity (8).

The mucosal-associated lymphoid tissues (MALTs) comprise the skin-associated lymphoid tissue (SALT), the gill-associated lymphoid tissue (GIALT), the gut-associated lymphoid tissue (GALT), and the recently described nasopharynx-associated lymphoid tissue. Common features of these MALTs resemble those of mammals and include the following: (i) a copious mucus layer that actively barriers pathogen adherence and agglutinates (9); (ii) secreted antimicrobial proteins (such as lysozyme, lectins, complement proteins, histones, and defensins), antibodies (*igm* and *igt*/*z* isotypes), immune mediators (cytokines and chemokines), and enzymatic disruptors (mainly proteases, peroxidases, and phosphatases); and (iii) interposed myeloid and lymphoid immune cells (including mast cells, dendritic-like cells, macrophages, neutrophils, and B and T lymphocyte families), natural killer cells (NK/NCC-like), epithelial phagocytic cells, and immune-associated cells such as thrombocytes and erythrocytes (6, 8, 10–12).

Skin-associated lymphoid tissue is the largest and most functionally diverse mucosal surface. Teleost skin is an extensive metabolically active non-keratinized multilayered integument that produces a complex glycoprotein-based mucus cuticle (8, 13) and also plays a crucial role in communication, sensory perception, locomotion, respiration, and osmoregulation (14, 15). Fish SALT harbors a more diverse repertoire of innate humoral components than the mammalian one, including bacteriolytic molecules such as lysozyme, complement components, lectins, proteolytic enzymes, C-reactive protein, interferons, and immunoglobulins (16). A whole cast of resident leukocyte families complete the immunological properties of SALT (8). The GIALT system is highly similar to that of skin and consists of interposed mucus-secreting cells, antimicrobial peptides, and resident leukocyte populations (11). Fish GALT lacks the mammalian Peyer’s patches, but presents intraepithelial lymphocytes that include T cells and some B cells located among epithelial cells. M-cell analogs and dendritic-like cells have also been described, as well as plasma cells, granulocytes, macrophages, and neuroendocrine cells inhabiting the epithelium or distributed in the lamina propria (17, 18). All these plethora of resources enable fish to defend from external agents, although it is not yet known how cells from gut recognize pathogenic bacteria among the commensal ones. But, if fish did not have this recognition system, the immune response in intestine would be active all the time because there are millions of commensals interacting with the epithelial cells.

Environmental or aquaculture-related insults couple the mucosal defensive reactivity with the activation of fish hypothalamic–pituitary–interrenal (HPI) and sympathoadrenomedullary (SAM) stress axis (19). As in the case of mucosal immune system, fish react differently depending on the stressor and the influence of the immune response on survival remains species specific (20). Plasmatic cortisol is a well-known indicator of stress situation experienced by fish (21) and also a recurrent mediator of bidirectional immunoendocrine regulation (22). In acute stressed fish, cortisol is secreted within several minutes up to 1 h into circulation (23). The release of cortisol from the head kidney modulates the leukocyte-mediated response and negotiates the onset, lag, and efficacy of immune reactivity. This may influence the mucous adherence and virulence of some pathogens (24) and may destabilize the host–microbiota interaction in favor of opportunistic pathogens (17). Little is known about the mechanisms of cortisol secretion in the mucosal tissues, but it has been suggested that cortisol levels in skin mucus correlate with those of plasma (24, 25) and may modulate specific tissue receptors and cytokine expression in mucosae (26). In this way, it has been reported that the stress-mediated increment of mucus-producing cells in mucosal surfaces induced a reduction in the number of parasites in mucosae (6, 27). Thus, fish mucosal barriers are thought to act as sensors playing a significant role in monitoring stress.

Thus far, the effects of stress on the immune system have been described mainly in systemic compartments including blood, head kidney, liver, and spleen. From these results, it has been implicitly assumed that the physiological stress response is similar among different fish species (21, 28). Moreover, little attention has been paid on the interaction and cross-modulatory effects between endocrine and immune systems among different fish species under stress situations. In fact, it has been recently reported that the combination of stress hormones and pathogen antigens could differentially induce a species-specific response (29). On the other hand, at the local response level, few studies have addressed the effects of stressful stimuli on the fish mucosal immune system. To date, several investigations have focused on the acute (25) and chronic (30) stress effects in mucosal tissues, but no study has elucidated the modulatory effect of different types of stressors (biotic, abiotic, and the combination of them) on mucosal tissues.

Although most fish show a generalized stress reaction *via* activation of primary and secondary responses (31), there is a specificity on the pattern and magnitude of the response that may be affected by not only environmental factors (such as temperature and salinity) but also the nature of the stressor. Our hypothesis was that fish respond qualitatively similar to stressors but that this response can be significantly modulated by both genetic background and environmental conditions. Therefore, we focused our work in the differential response between the two species.

In this study, we describe the short-term (1, 6, and 24 h) effect of a biotic stressor (*V. anguillarum* bacterin bath), an abiotic stressor (air exposure), and the combination of both stressors in physiological indicators (plasmatic and skin mucus cortisol) and SALT, GIALT, and GALT mRNA abundance (stress- and immune-related genes). These treatments were selected as similar handling procedures may be also often present when fish are subjected to vaccination in the aquaculture industry. This study was carried out using two commercial relevant species that inhabit in two distinct milieu: rainbow trout (*Oncorhynchus mykiss*; a freshwater teleost) and gilthead seabream (*Sparus aurata*; marine teleost). We aimed not only to clarify the role of mucosal immunity in the overall immune response of these two species under stress situations but also to show how the mucosae of aquatic vertebrates react to stressors of different nature.

## Materials and Methods

### Experimental Animals

Juveniles of rainbow trout (mean weight: 130 g) and gilthead seabream (mean weight: 65 g) were obtained from local fish farms (TroutFactory and Aquicultura els Alfacs, Spain) and acclimatized for 3 weeks at the Universitat Autònoma de Barcelona fish facility (AQUA-UAB) in conic tanks (2.0 m^3^ total capacity) with water pump, recirculating chiller cooling system, sand filter, and biofilter. Fish were maintained at a photoperiod of 12L:12D and at their respective environmental temperature (15°C for trout; 20°C for seabream). Fish were fed a commercial pellet (Skretting) at 1.5% of total body weight/day. Water quality indicators (dissolved oxygen, ammonia, nitrite, and pH) were analyzed periodically. These conditions were maintained also for the experimental tanks. The experiment complied with the Guiding Principles for Biomedical Research Involving Animals (EU2010/63), the guidelines of the Spanish laws (law 32/2007 and RD 53/2013), and authorized by the Ethical Committee of the Universitat Autònoma de Barcelona (Spain) for the use of laboratory animals.

### *V. anguillarum* Bacterin

An inactivated, formalin-killed *V. anguillarum*, serotype O1, O2α (the most pathogenic serogroup), and O2β, all with relative percentage survival ≥ 60% (Icthiovac^®^ VR, Hipra) was utilized as a source of antigen.

### Experimental Design

For the experiment, fish were placed in 300 l conic tanks with the closed recirculating system provided with water pump, sand filter, and biofilter. The temperature (15°C for trout; 20°C for seabream) and photoperiod (12L:12D) were set accordingly. Fish were divided into three groups and maintained in eight independent tanks. (1) *Vaccinated (v) group*: 48 fish were vaccinated by immersion (1 min) with formalin-killed *V. anguillarum* bacterin according to manufacturer’s instructions (Hipra). Immediately after, fish were rinsed in a cleaned water cube to discard the vaccine excess. Fish were then equally distributed (*n* = 12) in four tanks, avoiding cross-contamination for vaccine. (2) *Vaccinated and stressed (v* + *s) group*: 24 h after vaccination, 24 fish randomly selected from the vaccinated group were stressed (acute air exposure stress, 1 min) and returned to two separated tanks. (3) *Stressed (s) group*: 24 non-vaccinated fish were maintained out of water, stressed (acute air exposure stress, 1 min), and returned to two separated tanks. Control fish (*n* = 24) were mock-vaccinated (water vaccine-free immersion) in the same conditions as the vaccinated group, returned to two different separated tanks, and sampled after 24 h. Concerning time course utilized for the vaccine group, it should be stated that the preliminary data did not show any effect of vaccine immersion after 1 h and 12 h post vaccine (hpv) (data not shown). Therefore, we decided to begin sampling 24 h after bath vaccination. “Time 0” for vaccinated and vaccine + stress groups represents 24 hpv, whereas in the stress group represents the initial point of the experiment. Fish (*n* = 8) were randomly sampled from the two separated tanks per treatment at 1, 6, and 24 h post-stress (air exposure) from each experimental group (control, v, v + s, and s) and sacrificed by overanesthetization in MS222 (200 mg/l).

### Skin and Tissue Sampling

Rainbow trout and gilthead seabream skin mucus was sampled according to Xu et al. (32). After blood sampling, skin tissue samples (upper lateral line area behind the dorsal fin, left side, and roughly same size) were carefully taken to avoid muscle contamination. Gills (first lamella from both sides) were also sampled. For gut analysis, the body cavity was opened laterally, and midgut and hindgut were removed using a sterile scalpel and forceps. These harvested intestine sections were open longitudinally and feces and mucus carefully removed with forceps. Samples from all fish were immediately frozen in liquid nitrogen and stored at −80°C for further assays.

### Quantification of Cortisol in Plasma and Skin Mucus

Cortisol level was measured by radioimmunoassay (33), and the radioactivity was quantified using a liquid scintillation counter (Scintillation Counter Wallac 1409; PerkinElmer). Anti-cortisol antibody was used for the assay at the final dilution of 1:4,500. Antibody cross-reactivity with cortisol was 100%, and the lower detection limit of the assay was 0.16 ng/ml. Cross-reactivity with other steroid hormones varied from 1.6% for corticosterone and was inferior to 0.7% for other tested steroids.

### IgM Detection in Skin Mucus

Levels of IgM in rainbow trout and gilthead seabream skin mucus at 1, 6, and 24 h post-stress were determined by ELISA according to Cuesta et al with modifications (34). Rainbow trout skin mucus samples were 1/4 diluted in PBS + 10 mM EDTA. 50 µl/well was added and incubated at 4°C onto Maxisorp microplates (Thermo Fisher Scientific) in duplicate. The unbound antigen was removed by washing twice with 200 µl/well of PBS. Possible sites with no antigen bound were blocked with 100 µl/well non-fat milk 5% in PBS for 1 h at room temperature (RT) and washed twice with PBS. Antibody mouse anti-trout IgM 1.14 mAb (1/1,000 dilution in PBS) and anti-seabream IgM mAb (1/100 dilution in PBS determined by Western blot) (Aquatic Diagnostics Ltd., UK) were used as primary antibodies to detect the presence of IgM on skin mucus. Samples were incubated with 50 µl/well of primary antibody for 1 h at RT, followed by three times washing with 200 µl washing buffer (PBS + 0.15% Tween 20). Samples were incubated with 50 µl/well of goat-anti-mouse IgG conjugated with HRP (1/4,000 dilution in PBS). The microplate was washed five times with 200 µl/well of washing buffer, and 50 µl/well of Ultra-TMB (3,3′,5,5′-tetrametilbenzidine; Thermo Fisher Scientific) was added as a substrate. After incubation for 7 min at RT, 50 µl/well of H_2_SO_4_ (2 M) was added as stop solution and absorbance was determined at 450 (0.1 s) nm with a microplate reader (Victor3; Perkin Elmer). All samples were evaluated in duplicated.

### Isolation of RNA and cDNA Synthesis

Total RNA was isolated from individual fish samples using TRI reagent (Sigma) according to manufacturer’s instructions. The RNA pellet was dissolved in autoclaved milli Q-water and immediately stored at −80°C until use. The RNA concentration was quantified by a NanoDropND-2000 spectrophotometer (Thermo Fisher Scientific). Total RNA (2 µg) was used as a template to synthesize cDNA using High-Capacity cDNA Reverse Transcription Kits (Applied Biosystems) according to manufacturer’s instructions and immediately stored at −20°C until use.

### Quantitative Real-Time PCR

Fish mucosal samples including skin, gills, and gut were analyzed using real-time PCR. The analysis included the evaluation of stress and immune-related genes (*lysozyme, c3, igm, hsp70, cox2, il1β, tnfα, il6, tgfβ1*, and *il10*). We tested several housekeeping candidate genes in rainbow trout (*ef1α* and *βactin*) and seabream (*18s, ef1α*, and *rpl27*) to elucidate which one had less variation. *β-Actin* (for rainbow trout) and *18s* (for seabream) were included on gene expression analysis. Specific primers used for rainbow trout (Table 1) and gilthead seabream (Table 2) are indicated. Primers were designed with Primer-Blast. The primer secondary structure and annealing specificity was checked with OligoAnalyzer (version 3.1) and Primer-Blast software, respectively. The undesirable PCR products appearance was previously verified by single peak in the melting curve for each primer set. The primer amplification efficiency was determined in all mucosal surfaces included in our study. Real-time PCR reactions were performed with iTaq universal sybr green supermix (Bio-Rad Laboratories) using 1:20 and 1:10 cDNA dilution made for genes of interest in rainbow trout and gilthead seabream, respectively. Primers for all genes were used at a final concentration of 500 nM. The thermal conditions used were 3 min at 95°C of pre-incubation followed by 40 cycles at 95°C for 30 s and 60°C for 30 s. All the reactions were performed in duplicate using CFX384 Touch Real-Time PCR Detection System (Bio-Rad Laboratories). Quantification was done according to the Pfaffl method (35) corrected for efficiency of each primer set obtained for each mucosal surface evaluated. Values for each experimental condition were expressed as normalized relative expression against those of the housekeeping gene *β-actin* and 18s for rainbow trout and seabream, respectively. Results are expressed as average of values obtained for the same treatment and time points evaluated.

**Table 1:** Primers used for gene expression analysis in rainbow trout.

**Gene**	**GenBank accession number**	**Sequence 5′–3′**	**Product size**	**Primer efficiency**
*βactin*	NM_001124235.1	FW: GGACTTTGAGCAGGAGATGG	186	1.96
		RV: ATGATGGAGTTGTAGGTGGTCT		

*lys*	X59491	FW: TGCCTGTCAAAATGGGAGTC	211	1.89
		RV: CAGCGGATACCACAGACGTT		

*c3*	L24433	FW: GAGATGGCCTCCAAGAAGATAGAA	91	1.96
		RV: ACCGCATGTACGCATCATCA		

*igm*	S63348.1	FW: AAGAAAGCCTACAAGAGGGAGA	157	1.85
		RV: CGTCAACAAGCCAAGCCACTA		

*hsp70*	AB176854	FW: CGGGAGTTGTAGCGATGAGA	140	2.01
		RV: CTTCCTAAATAGCACTGAGCCATAA		

*cox2*	NM_001124348.1	FW: AGCACTTCACCCACCAGTTC	180	1.85
		RV: GGTAGACCTCGCCGTTCAAA		

*il1β*	NM_001124347.2	FW: TGAGAACAAGTGCTGGGTCC	148	1.92
		RV: GGCTACAGGTCTGGCTTCAG		

*tnfα*	NM_001124357.1	FW: CACACTGGGCTCTTCTTCGT	155	1.88
		RV: CAAACTGACCTTACCCCGCT		

*il6*	NM_001124657.1	FW: GAGTTTCAGAAGCCCGTGGA	149	2.04
		RV: AGCTGGTACACTTGCAGACC		

*tgfβ1*	NM_001281366.1	FW: GCCAAGGAGGTCCACAAGTT	146	1.94
		RV: GTGGTTTTGATGAGCAGGCG		

*il10*	NM_001245099.1	FW: CCGCCATGAACAACAGAACA	105	1.91
		RV: TCCTGCATTGGACGATCTCT		

**Table 2:** Primers used for gene expression analysis in gilthead seabream.

**Gene**	**GenBank accession number**	**Sequence 5′–3′**	**Product size**	**Primer efficiency**

*18s*	AY587263.1	FW: ACCAGACAAATCGCTCCACC	172	2.02
		RV: AGGAATTGACGGAAGGGCAC		

*lys*	AM749959.1	FW: TCATCGCTGCCATCATCTCC	154	2.08
		RV: TGTTCCTCACTGTCCCATGC		

*c3*	HM543456.1	FW: GTTCCACAACAACCCACAGC	183	1.91
		RV: ACATACGCCATCCCATCCAC		

*igm*	JQ811851.1	FW: GATCGTGACATCGTCTGAGG	187	1.91
		RV: TGTTGGGTTGTGGTTGTAGG		

*hsp70*	EU805481.1	FW: AGGTTGGGTCTGAAAGGAAC	174	1.96
		RV: TGAACTCTGCGATGAAGTGG		

*cox2*	AM296029.1	FW: GAGTACTGGAAGCCGAGCAC	192	1.89
		RV: GATATCACTGCCGCCTGAGT		

*il1β*	AJ277166.2	FW: TCAGCACCGCAGAAGAAAAC	115	1.97
		RV: TAACACTCTCCACCCTCCAC		

*tnfα*	AJ413189.2	FW: TCGTTCAGAGTCTCCTGCAG	320	2.24
		RV: AAGAATTCTTAAAGTGCAAACACACCAAA		

*il6*	EU244588.1	FW: ATCCCCTCACTTCCAGCAGA	129	1.86
		RV: GCTCTTCGGCTCCTCTTTCT		

*tgfβ1*	AF424703.1	FW: AGACCCTTCAGAACTGGCTC	145	1.90
		RV: ACTGCTTTGTCTCCCCTACC		

*il10*	JX976621.1	FW: GAGCGTGGAGGAATCTTTCAA	154	2.02
		RV: GATCTGCTGGATGGACTGC		

### Statistical Analysis

The statistical package for social science (SPSS, v20) software was used for the analysis. The Generalized Linear Model was utilized considering the stressors and time dynamics as a two between-subjects factor. This model is a more flexible statistical tool than the standard general linear model in terms of types of distribution and different covariance structure of the repeated measures, does not require homogeneity of variance, and it admits missing values. After the main analysis, appropriate pair-wise comparisons were carried out. Differences in all data were considered statistically significant if *p*-values < 0.05 among groups.

## Results

### Plasmatic and Skin Mucus Cortisol Level

**Figure 1:**
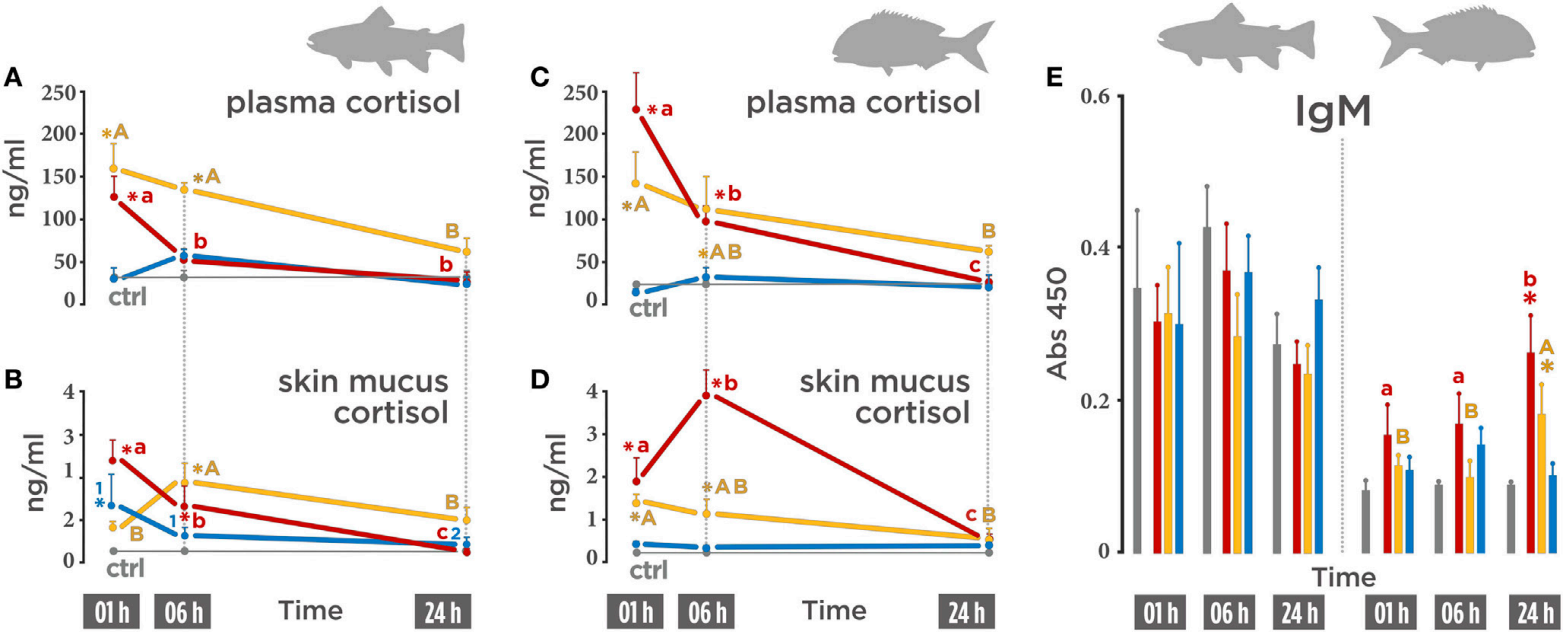
Cortisol and IgM levels on biotic (*Vibrio anguillarum* bacterin), abiotic (air exposure), or the combination of both stressors (vaccine + air exposure) at 1, 6, and 24 h post-stress. **(A)** Rainbow trout plasmatic cortisol (ng/ml). **(B)** Rainbow trout skin mucus (ng/ml) cortisol. **(C)** Gilthead seabream plasmatic cortisol. **(D)** Gilthead seabream skin mucus cortisol. **(E)** Total IgM (Abs 450) in rainbow trout (left side) and seabream (right side). Different colors indicate different treatments: red (air exposure), orange (vaccine + air exposure), blue (vaccine), and gray (ctrl; control). Time zero represents the end of the acute air exposure stress (for the air exposure and the vaccine + air exposure) or 24 h after bath vaccination (for vaccine group). Data are represented as mean ± SE (*n* = 8 per sampling time point). Significant differences are indicated by lower case in the air exposure group, by upper case in the vaccine + air exposure group, and by numbers in the vaccine group. Asterisk (*) indicates significant difference of each treatment versus control (*p* < 0.05).

In order to evaluate whether the application of air exposure, *V. anguillarum* bacterin, or the combination of both stressors induce a stress response systemic (plasmatic) and local (skin mucus), cortisol levels were evaluated. In trout, plasmatic cortisol levels augmented after air exposure (126.32 ng/ml) 1 h post-stress (Figure 1A; red line) and decreased at 6 h post-stress (49.57 ng/ml). The highest cortisol concentration was registered in the vaccine + air exposure group (159.85 ng/ml) at 1 h post-stress (Figure 1A; orange line). A slight decrease, although still higher than the control group, was also observed at 6 h post-stress in the vaccine + air exposure group (135.40 ng/ml). In the vaccinated group, no variations were registered on plasma cortisol (Figure 1A; blue line).

The same secretion pattern was observed in skin mucus in the air exposure group. Cortisol levels augmented at 1 h post-stress (2.41 ng/ml), diminished at 6 h post-stress (1.34 ng/ml), and returned to control level at 24 h post-stress (0.20 ng/ml) (Figure 1B; red line). In the vaccine + air exposure group, an increase in cortisol was only observed at 6 h post-stress (1.99 ng/ml) (Figure 1B; orange line). In the vaccinated group, cortisol was significantly increased at 1 h post-stress (1.48 ng/ml) (25 h after vaccination) and recovered baseline values at 24 h post-stress (48 h after vaccination) (Figure 1B; blue line).

In gilthead seabream, the secretion patterns were similar but differed in magnitude. Significantly elevated plasma cortisol levels at 1 h post-stress (228.18 ng/ml) (Figure 1C; red line) were detected in the air exposure group, decreased at 6 h post-stress (96.40 ng/ml) and dropped to control levels at 24 h post-stress (25.71 ng/ml). In the vaccinated + air exposure group, the cortisol values were higher than control at 1 h (144.08 ng/ml) and 6 h (113.30 ng/ml) but not at 24 h post-stress (61.0 ng/ml) (Figure 1C; orange line). The vaccinated seabream group showed no variations (Figure 1C; blue line).

Cortisol levels in seabream skin mucus differed from those observed in trout skin mucus. High cortisol levels (1.89 ng/ml) were registered in the air exposure group at 1 h post-stress (Figure 1D; red line) and doubled at 6 h post-stress (3.91 ng/ml). In the vaccinated + air exposure group, cortisol levels augmented at 1 h (1.43 ng/ml) and 6 h post-stress (1.18 ng/ml) (Figure 1D; orange line). No significant variations were observed in the vaccinated group (Figure 1D; blue line).

The differences noted between trout and seabream skin mucus were also observed when the total amount of IgM was determined by ELISA. In trout skin mucus, the levels of IgM showed no variations after the different treatments (Figure 1E; left half). However, in seabream the levels of IgM in the air exposure group increased gradually, reaching a peak at 24 h post-stress (Abs_450_ = 0.25) compared to control (Figure 1E; right half). This augment was also registered at 24 h post-stress in the vaccinated + air exposure group (Abs_450_ = 0.17) (Figure 1E; right half).

Thus, our results indicate that the release of the glucocorticoid hormone in response to stressor depends on the stressor, the biological matrix (plasma or mucus), and is species dependent.

### SALT Responses

**Figure 2:**
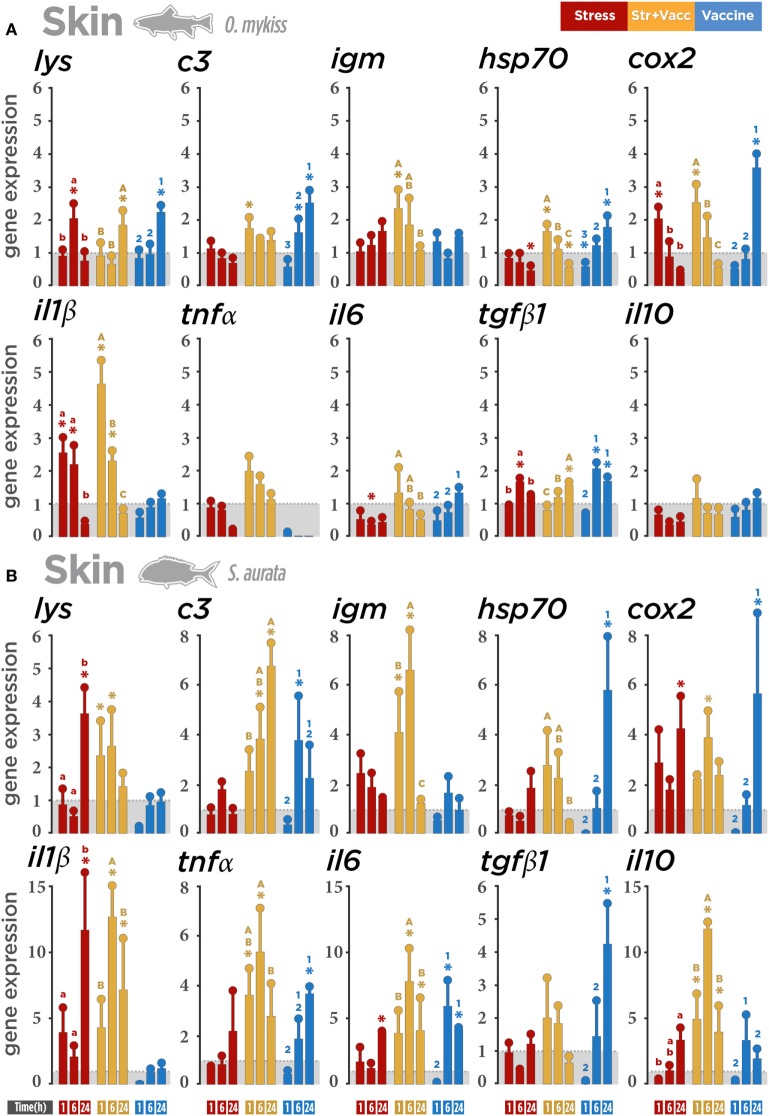

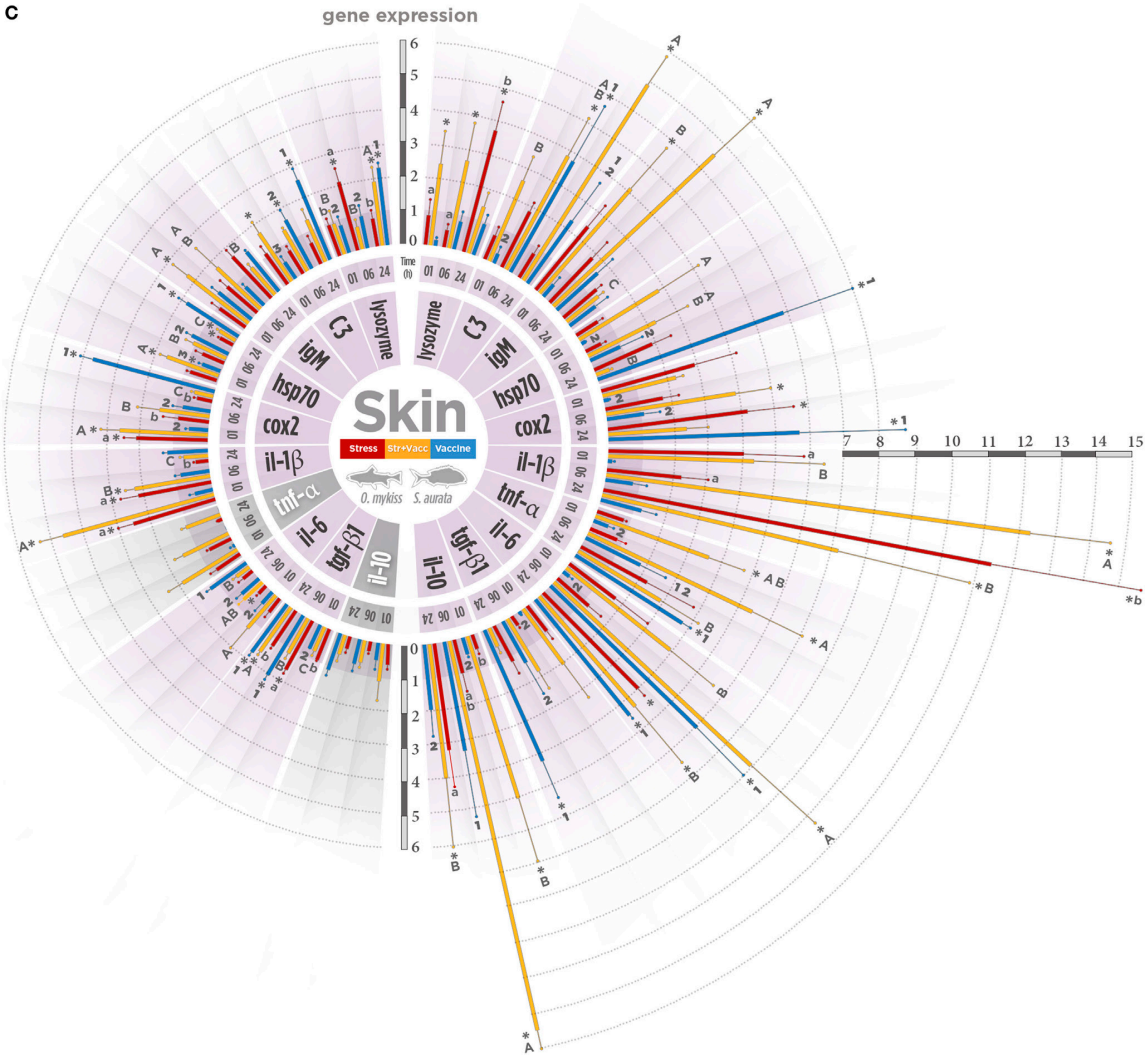
Gene expression level in skin-associated lymphoid tissue (SALT) after a biotic (*Vibrio anguillarum* bacterin), abiotic (air exposure), or the combination of both stressors (vaccine + air exposure) at 1, 6, and 24 h post-stress. Quantitative real-time PCR quantification of specific mRNA immune and stress transcripts (*lysozyme, c3, igm, hsp70, cox2, Il1β, tnfα, il6, tgfβ1*, and *il10*) in **(A)** rainbow trout and **(B)** gilthead seabream skin. **(C)** Integrative comparison of SALT transcriptomic responses. *β-Actin* and *18s* were chosen as housekeeping genes in rainbow trout and gilthead seabream, respectively. Different colors indicate different treatments: red (air exposure), orange (vaccine + air exposure), blue (vaccine), and gray (ctrl). Time zero represents the end of the acute air exposure stress (for the air exposure and the vaccine + air exposure) or 24 h after bath vaccination (for vaccine group). Data are represented as mean ± SE (*n* = 8 per sampling time point). Significant differences are indicated by lower case in the air exposure group, by upper case in the vaccine + air exposure group, and by numbers in the vaccine group. Asterisk (*) indicates significant difference of each treatment versus control (*p* < 0.05).

mRNA expression levels were used to examine whether the air exposure as well as *V. anguillarum* were able to drive differences in the transcriptomic responses of stress and immune-related genes in MALTs: skin, gills, and gut. Our results show an overall significant interaction between treatment and time course at 1, 6, and 24 h post-stress in both species. In rainbow trout (Figure 2A), air exposure was able to enhance the transcription of *il1β, cox2*, and *lysozyme* in a time-dependent manner. The vaccine + air exposure treatment promoted the upregulation of genes associated with immunity and stress responses (*c3, igm, hsp70*, and *cox2*) at 1 h post-stress. The expression of pro-inflammatory transcripts (*il1β*) was also upregulated. The same effect was also observed at 6 h post-stress for *il1β* but not for *cox2*. No modulation was observed at 1 h post-stress in gene transcripts associated with anti-inflammatory responses (*il10* and *tgfβ1*). However, the upregulation of *tgfβ1* was only observed at 6 and 24 h post-stress in the vaccinated group, probably linked with the upregulation also observed for *lysozyme, c3, cox2*, and *hsp70*. Overall, all genes showed a marked upregulation in a treatment- and time-independent manner.

In gilthead seabream (Figure 2B), air exposure was found to induce pro-inflammatory cytokine transcripts (*il1β* and *il6*), *cox2*, and also *lysozyme* at 24 h post-stress. The upregulation of genes in the vaccine + air exposure group was also observed, though the magnitude and time course of this modulation was shown to be different compared to rainbow trout. A high and decreasing expression from 1 h post-stress to 24 h post-stress in *hsp70* and *lysozyme* was reported. The same expression pattern was observed for *tgfβ1*. An increased gene expression at 6 h post-stress was noted for *il1β, il6, tnfα, cox2*, and *igm*. The upregulation of *il10* was modulated in the same manner. An increase in a time-dependent manner was registered only for *c3*. Importantly, this upregulation in the vaccine + air exposure group seems to be influenced by air exposure and vaccine separately. Only in the cases of *il1β* and *il6*, the effect observed in the vaccine + air exposure group could be markedly associated with the expression registered in the air exposure group and vaccinated group, respectively. Importantly, *V. anguillarum* bacterin was able to induce expression of *il6, tnfα, tgfβ1, hsp70, cox2*, and *c3* mainly at 24 h post-stress.

In summary, a lower gene expression magnitude was observed in rainbow trout than in seabream (Figure 2C). In contrast to rainbow trout response, the gene expression data suggest a higher influence of the air exposure stressor and the combination of both stimuli in gilthead seabream. The similar expression of *lysozyme, hsp70*, and *tgfβ1* suggests that the anti-inflammatory cytokine response could modulate the expression of these immune-related genes in both species when stressed.

### GIALT Responses

**Figure 3:**
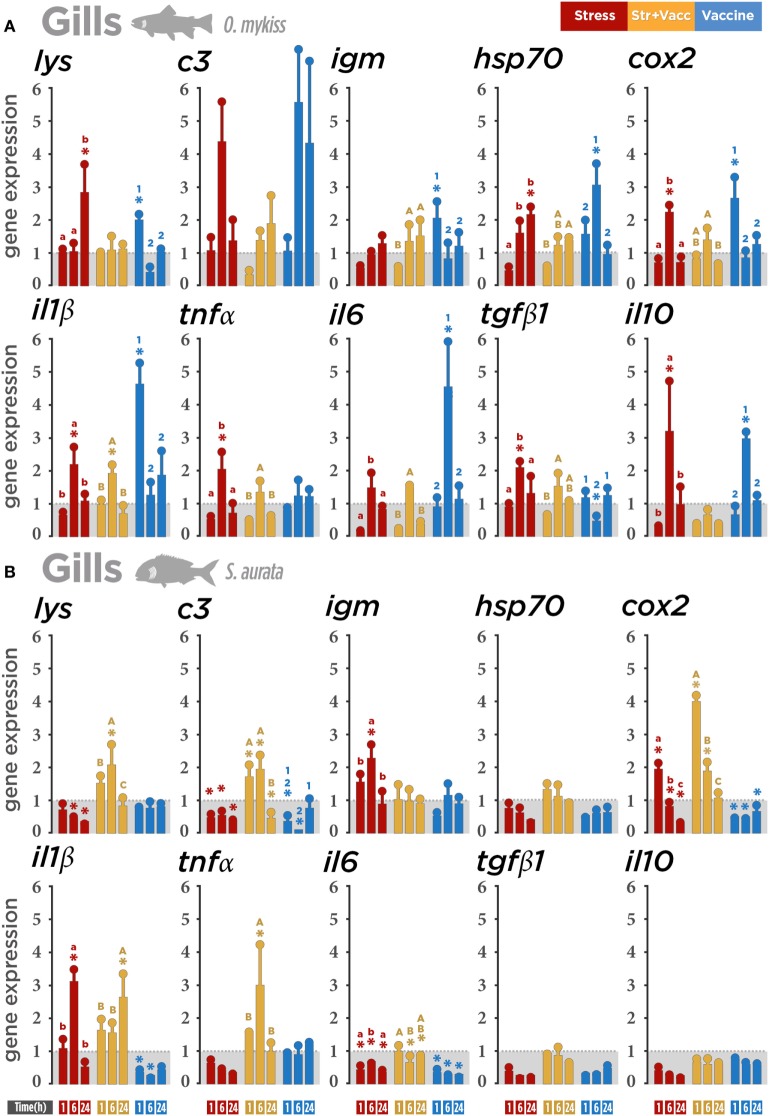

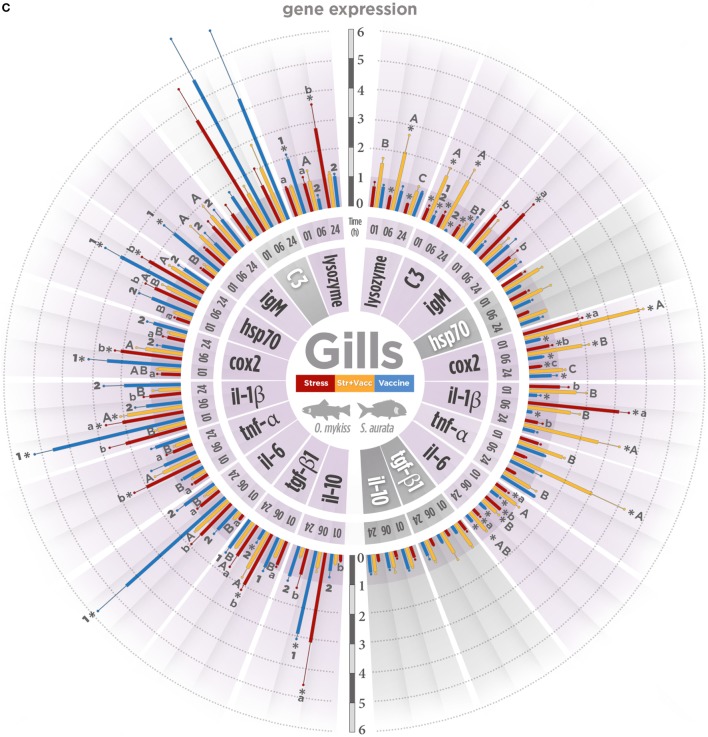
Gene expression level in gill-associated lymphoid tissue (GIALT) after a biotic (*Vibrio anguillarum* bacterin), abiotic (air exposure), or the combination of both stressors (vaccine + air exposure) at 1, 6, and 24 h post-stress. Quantitative real-time PCR quantification of specific mRNA immune and stress transcripts (*lysozyme, c3, igm, hsp70, cox2, Il1β, tnfα, il6, tgfβ1*, and *il10*) in **(A)** rainbow trout and **(B)** gilthead seabream gills. **(C)** Integrative comparison of GIALT transcriptomic responses. *β-Actin* and *18s* were chosen as housekeeping genes in rainbow trout and gilthead seabream, respectively. Different colors indicate different treatments: red (air exposure), orange (vaccine + air exposure), blue (vaccine), and gray (ctrl; control). Time zero represents the end of the acute air exposure stress (for the air exposure and the vaccine + air exposure) or 24 h after bath vaccination (for vaccine group). Data are represented as mean ± SE (*n* = 8 per sampling time point). Significant differences are indicated by lower case in the air exposure group, by upper case in the vaccine + air exposure group, and by numbers in the vaccine group. Asterisk (*) indicates significant difference of each treatment versus control (*p* < 0.05).

Gills showed a different gene expression pattern when comparing both fish species. In trout (Figure 3A), the gene transcript modulation in the air exposure group was observed at 6 h post-stress in pro- (*il1β* and *tnfα*), anti-inflammatory genes (*il10* and *tgfβ1*), and *cox2*. Particularly, the downregulatory tendency was observed in the air exposure group and the vaccine + air exposure group at 1 h post-stress, suggesting that the stress by air exposure could influence the early post-stress expression of trout transcripts. In the vaccinated group, the expression of *il1β* and *cox-2, lysozyme*, and *igm* were observed at 1 h post-stress. At 6 h post-stress, the expression levels of *il6* and *hsp70* were also modulated. Importantly, *il10* was also upregulated at 6 h post-stress, suggesting that its modulation could be related to the control of the pro-inflammatory gene expression profile. This suggests that air exposure and vaccine alone had a stronger effect on gene expression in trout.

In seabream (Figure 3B), a marked upregulation of the expression of *il1β, tnfα, cox2, lysozyme*, and *c3* was registered both at 1 and 6 h post-stress and after vaccine + air exposure. The upregulation of *cox2* (1 h post-stress) and *il1β* (6 h post-stress) suggests a specific gene expression effect of air exposure in seabream. The downregulation of several genes in the air exposure and vaccinated groups suggests that the stress stimuli including air exposure and *V. anguillarum bacterin* alone may suppress seabream immune response in gills.

Our results indicate that, aside from the increase in few genes in the gills of rainbow trout and seabream, both stressors separately induce immune suppression or a tendency to reduce immune and stress gene transcription in the gills (**Figure 3C**).

### GALT Responses

**Figure 4:**
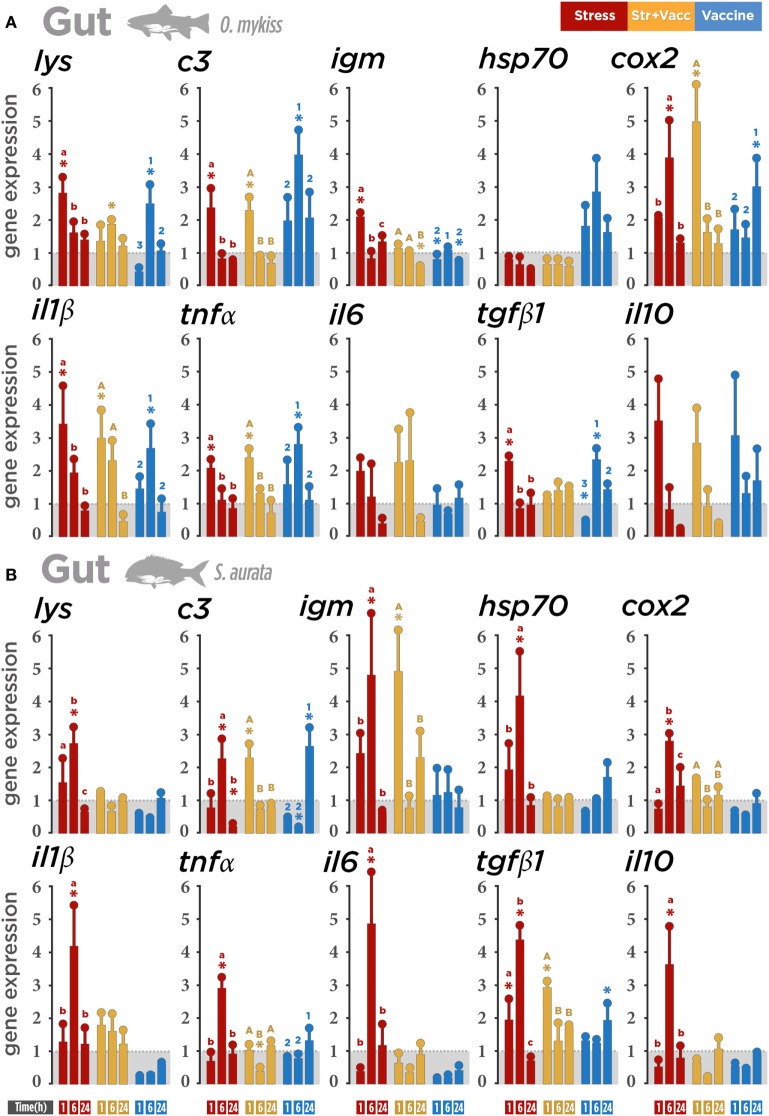

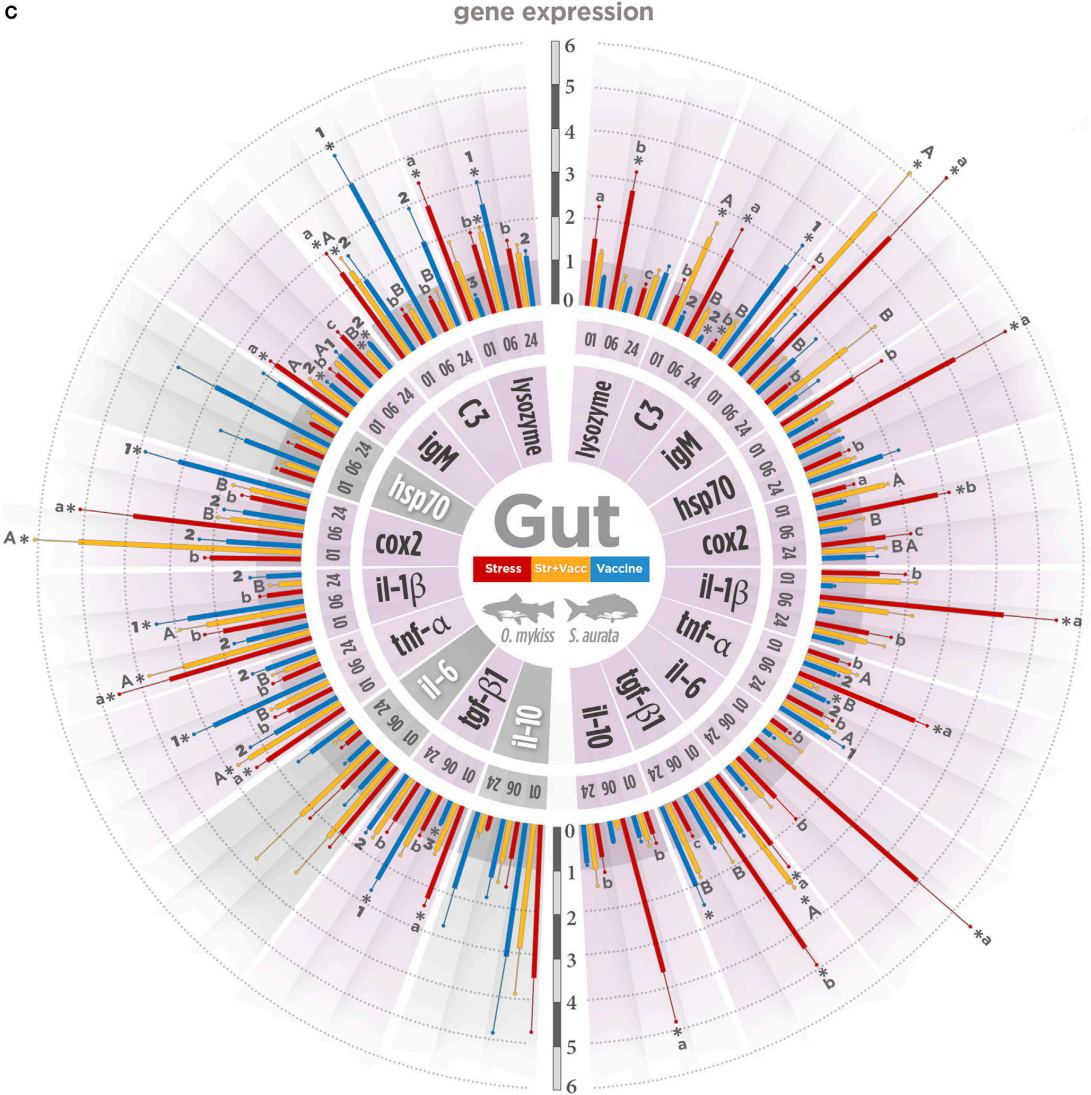
Gene expression level in gut-associated lymphoid tissue (GALT) after a biotic (*Vibrio anguillarum* bacterin), abiotic (air exposure), or the combination of both stressors (vaccine + air exposure) at 1, 6, and 24 h post-stress. Quantitative real-time PCR quantification of specific mRNA immune and stress transcripts (*lysozyme, c3, igm, hsp70, cox2, Il1β, tnfα, il6, tgfβ1*, and *il10*) in **(A)** rainbow trout and **(B)** gilthead seabream. **(C)** Integrative comparison of GALT transcriptomic responses. *β-Actin* and *18s* were chosen as housekeeping genes in rainbow trout and gilthead seabream, respectively. Different colors indicate different treatments: red (air exposure), orange (vaccine + air exposure), blue (vaccine), and gray (ctrl; control). Time zero represents the end of the acute air exposure stress (for the air exposure and the vaccine + air exposure) or 24 h after bath vaccination (for vaccine group). Data are represented as mean ± SE (*n* = 8 per sampling time point). Significant differences are indicated by lower case in the air exposure group, by upper case in the vaccine + air exposure group, and by numbers in the vaccine group. Asterisk (*) indicates significant difference of each treatment versus control (*p* < 0.05).

Transcriptomic profile analysis of both species showed that air exposure modulated the immune- and stress-related gene expression transcripts in gut. In trout (Figure 4A), the upregulation of gene transcripts involved in regulatory responses (*lysozyme, c3, il1β, tnfα, tgfβ1, cox2*, and *igm*) was observed at 1 h post-stress indicating that, in gut, air exposure induces the upregulation of immune-related genes in trout earlier than in seabream. The same modulation of the pro-inflammatory genes (*il1β* and *tnfα*) was also observed in trout at 1 h post-stress in the vaccine + air exposure group, suggesting that this response could be directly influenced by the air exposure at the same time-point. *il1β, tnfα, tgfβ1, c3*, and *lysozyme* were upregulated in the vaccinated group after 6 h post-stress, *cox2* after 24 h post-stress, and *igm* remained downregulated.

Interestingly, in seabream gut (Figure 4B), the larger alteration was induced mainly by air exposure. The transcriptional level of immune and stress regulators (*il1β, il6, tnfα, il10, tgfβ1, cox2, hsp70, lysozyme, c3*, and *igm*) was enhanced at 6 h post-stress, indicating a higher sensitivity of this specie to air exposure. Our results suggest that in gut the air exposure stress promotes a roughly similar immune-related gene expression modulation in both species although in a different magnitude and time-dependent manner (Figure 4C).

Taken together, the results show that both stressors modulate the SALT, GIALT, and GALT transcriptomic response, but such response depends on the nature of the stressor, time, and the species concerned.

## Discussion

In this study, we aimed to evaluate the isolated and combined effects of a biotic (*V. anguillarum* bacterin bath) stressor and abiotic (air exposure) on SALT, GIALT, and GALT of two different species, trout and seabream. To do so, we analyzed the gene expression patterns of several relevant stress- and immune-related transcripts (*lysozyme, c3, igm, hsp70, cox2, Il1β, tnfα, il6, il10*, and *tgfβ1*) and plasmatic and mucosal (skin mucus) cortisol levels. Overall, our results indicate that short-term stressors modify plasma and skin cortisol levels and regulate the transcriptomic response of several immune mediators at mucosal surfaces in both species. The observed changes suggest that stress disturbs the mucosal tissue and mainly enhances the mucosal immune response in a pronounced species-specific manner. Contrary to the notion that stress suppresses the immune response, we clearly show that under certain conditions, particularly short-term disturbances, stress can activate mucosal immune function.

### SALT Responses

Once a stressor is sensed by the host, the activation of the HPI-SAM axis releases cortisol, a stress biomarker that, in turn, activates secondary and tertiary stress responses (36). It should be taken into account that an accurate description of the integrated stress mechanisms should include the role of local stress and immunoregulatory responses (31) such as those of mucosal surfaces. The mucosal immune system is considered an active immunological interface (4) in which stressors of different scale and sources may recruit HPI-SAM axis elements and impose long-term disturbances (21, 37, 38). Skin is one of the mucosal immune tissues that act as first barrier against both pathogens and stressors (6). Interestingly, recent work on the local stress response in skin involved the cortisol measurement in scales (39) and also in skin mucus (25), as the cortisol lipophilic nature makes feasible its diffusion through cell membranes. However, the mechanisms of mucus cortisol presence are poorly understood (25), but the amount of cortisol in mucosal surfaces may be a proxy for plasmatic cortisol levels or an indirect indication of local inflammation. In fact, recent research showed genes and peptides in peripheral tissues related to molecules of the stress axis (40). Therefore, such an issue is far from having a definitive answer, and more research has to be done in this matter.

In this study, cortisol levels following air exposure peaked in a time-dependent and species-specific manner. The higher cortisol levels after stress in gilthead seabream may indicate a lower activation threshold to air exposure. This effect did not take place after *V. anguillarum* exposure. This differential activation threshold would imply a higher activity of interrenal cells, as it was already proposed (41). While the cortisol levels in the vaccine + air exposure group were higher than the levels observed in the air exposure group in trout, in seabream an opposite situation was true, suggesting a higher sensitivity to the combination of stressors on HPI activation in rainbow trout.

Concerning *V. anguillarum* bacterin treatment, cortisol skin mucus levels raised only in trout. It has been shown that common fish pathogens such as *Lepeophtheirus salmonis, Edwardsiella ictaluri, V. anguillarum, Aeromonas salmonicida*, and *Pseudomonas anguilliseptica* are capable of inducing cortisol release in fish (42–45). Particularly, *V. anguillarum* is a widespread fish pathogen that adheres to the mucosal tissues and elicits a strong cortisol-mediated stress response (46). Based on the results obtained in this study, it seems that the differential effect to *V. anguillarum* may either reside on a differential response at the mucosal level among the two species or the antigenic effect causing endocrine alterations. As stated before, the highest plasmatic cortisol values were registered in the vaccine + air exposure group for trout but not for seabream. Therefore, the apparently higher reactivity to the stimuli observed in trout skin mucus could help to explain the highest plasmatic cortisol levels obtained in trout enduring the combination of stressors. By contrast, in seabream it seems that cortisol increase was produced only in the air exposure treatment.

It has been reported that higher levels of cortisol and catecholamines following exposure to acute stressors may increase the number of circulating leukocytes, specifically neutrophils, and reduce lymphocyte numbers (21, 47–49). Thus, a modulation in IgM levels during acute stress responses is to be expected, although no alterations in the seric IgM levels in seabream after acute air exposure stress was reported in previous work (34). IgM is the most abundant immunoglobulin in skin mucus and provides protection against pathogens that are in close contact with outermost fish surfaces (6). In this study, no changes were observed in any of the stressors tested for IgM trout skin mucus. Data also indicate that the augment of cortisol on seabream skin mucus registered at 1 and 6 h post-stress did not modify the levels of IgM. However, at 24 h post-stress, the air exposure and the combination of the vaccine + air exposure stressors (not vaccinated) were able to increase the amount of total IgM in seabream skin mucus. The increase in IgM skin mucus could be associated with an immune protective mechanism in response mainly to the air exposure stress in seabream, reinforcing the hypothesis of a lower activation threshold to acute handling stress in seabream.

In this study, skin shows the highest transcript abundance of all three mucosal surfaces, particularly in seabream. This upregulatory response of trout skin was mainly observed in the vaccine + air exposure and in the vaccinated groups. By contrast, the increase in transcript expression in seabream skin was observed particularly in the air exposure and vaccine + air exposure groups, again suggesting a higher responsiveness to biotic stimuli in trout and to abiotic stimuli in seabream. *cox2* mRNA levels were elevated in skin of trout and at a greater extent in seabream, in agreement with the previously described increase in *cox2* after acute stress in the skin and intestine (37, 50). The upregulation of *lysozyme, c3*, and *igm* in trout and seabream vaccine + air exposure groups indicates that the combination of different stressors may activate the mucosal immunity. *Lysozyme* and *c3* are ubiquitously expressed antimicrobial and bactericidal components of the innate arm of the mucous immune system (51), and expression of *c3* indicates that extrahepatic *c3* also may play a role in stress-mediated local mucosal immunity responses. The expression of seabream IgM on skin mucus was not correlated with the seabream expression pattern on skin. However, it is important to take into consideration that, although directly related, skin mucus and skin are considered different tissue matrices as also suggested by their distinct role in the stress response. While in the skin the modulation of gene expression takes place mostly in resident cells, in skin mucus the total protein content could be influenced not only by the skin resident cells but also by the cell trafficking and protein secretion as an outcome of stress responses. Due to the intimate contact with the surrounding environment, the provoked immune response in the skin may activate a local alert for the endocrine messengers in the mucosa to be prepared for potential challenges. It is worthy to note that during acute stress, skin is enriched in leukocytes and, as it was stated before, short-term stress substantially increases trafficking of leukocytes to the skin in mammals and fish (52, 53), assuring the mobilization of leukocytes to skin and probably the IgM synthesis.

The inflammatory response plays a key role in the host defense activation mechanisms. Not only pro- but also anti-inflammatory cytokine transcriptions were dramatically raised in seabream skin. This suggests an attempt to control/unleash a nascent inflammatory response, recruiting anti-inflammatory and wound-healing agents such as *il10* and *tgfβ1* (54, 55). Analog to mammals, fish inflammatory responses are characterized by a first wave of expression of pro-inflammatory cytokines (56–58). At later stages of inflammation, the release of a second wave of anti-inflammatory cytokines by macrophages initiates the process of recovery, which is pivotal to reduce the inflammation (57). Moreover, the excessive induction of the pro-inflammatory agents and innate immune components may not only harm the host but also impose more energy consumption (59). Therefore, this mechanism could also be related to minimize the energy expenditure in other physiological processes different from the stress response.

In sum, a differential modulatory effect affecting the mRNA abundance of relevant immune biomarkers was determined in skin. Particularly, the seabream response was characterized by a significant upregulation on genes related to immune and stress response to air exposure and the combination of vaccine and air exposure stimulus.

### GIALT Responses

Several pathogens show a preference for gills during the adherence phase of the infective cycle. In this way, it has reported that a pathogen is able to rapidly modify the host mucus transcriptomic responses to facilitate bacterial adherence (58). *V. anguillarum* has been shown to cause serious diseases in fish gills provoking upregulation of both pro-inflammatory cytokines and their mediator molecules in trout and cod (60–62). Our results with *V. anguillarum* bacterin show similar responses among mucosae but a clear difference in terms of gene expression between species. In gill mucosa, the inflammatory response triggered by stressors showed a general suppression of transcripts in seabream but not in trout. The increase in transcription of pro-inflammatory cytokines *il1β* and *il6*, simultaneously with the *il10* increment following vaccine exposure, indicates that inflammation would be the predominant response in trout. Thus, markers of bacteriolytic responses in mucosal surfaces such as *lysozyme* and complement component *c3* (63, 64) were upregulated in trout but not in seabream, therefore, providing more arguments to the modulatory gene activation of immunity-related genes to *V. anguillarum* bacterin in trout. The same modulatory effect by *V. anguillarum* bacterin treatment in trout gills and the air exposure stressor in seabream was also observed in the *hsp70* and *cox2* transcript levels. This clear distinct direction of the gill response found between trout and seabream, in addition to the specificity of the response, may be associated with their difference in the genetic diversity and also with the environmental conditions such as temperature. Our results show that a most marked downregulation in seabream occurs after both air exposure and vaccine stressors. Compared to skin and gut, the overall lower expression values of immune- and stress-related genes indicate that the gill mucosa seems to be less responsive or more regulated after stress. Therefore, notwithstanding the upregulation of gene expression in trout (*il1β, il6*, and *il10*) to *V. anguillarum* bacterin, the overall response to stressors in the branchial tissue may obey to the constraints of the metabolic trade-offs between respiratory, osmoregulatory, and immune processes in such a multifunctional organ that may confine the number of resident macrophages and lymphocytes.

### GALT Responses

Gut not only carries out the nutrient absorption but also acts as a physical and chemical barrier in which innate and adaptive immune responses are also crucial for protection (4, 10, 65, 66). Unlike the expression observed in trout skin and gills, a mixed effect was observed in trout gut depending on the gene evaluated. According to the results obtained in trout skin mucus, skin, and gills, an influence on the gene modulation (particularly *lysozyme* and *igm*) by vaccine + air exposure and vaccine groups was registered. However, in trout gut a similar expression pattern (*c3, il1β*, and *tnfα*) was observed in fish subjected to air exposure and vaccine + air exposure. *Lysozyme* expression in rainbow trout intestine agrees with previous results obtained in Atlantic cod (*Gadus morhua*) vaccinated against *V. anguillarum*, showing induction of antibacterial genes (61). This indicates, contrary to the expression of lysozyme found in skin, that the modulation of intestinal *lysozyme* expression by stressors may be tissue and/or species dependent. The upregulation of *cox2* is consistent with previous reports in Atlantic salmon showing increments of *cox2a* in midgut 1 h after stress (50, 67). On the other hand, impairment of intestinal functions has also been observed in mammals as a consequence of prostaglandin increment. As it has been previously described, cortisol-mediated stress responses may alter intestinal permeability (68), hence animals prevent such an increment of permeability through reduction of the prostanoid content after acute stress, which confirms the impact of prostaglandins on intestinal homeostasis (67) in connection with the expression of *cox2* induced by *il1β* expression (69). Therefore, the modulatory effect of *cox2* observed in this study for both species could either be explained as a result of inflammation or cortisol elevation in fish.

The expression of trout pro/anti-inflammatory (*il1β, tnfα*, and *tgfβ1*) cytokines was mainly enhanced by air exposure and vaccination in gut, while mRNA abundance of seabream cytokines (*il1β, il6, tnfα, il10*, and *tgfβ1*) was mainly induced by air exposure. As mentioned before, the balance between pro- and anti-inflammatory cytokines is crucial to control the inflammation. Previously reported gene expression levels of *il1β* in Atlantic salmon intestine decreased after 7 weeks exposure to hypoxia, suggesting that short-term and long-term stress may induce differential regulation of cytokines (70, 71). *igm* mRNA abundance was stimulated in trout and seabream after air exposure at different time points. The data suggest that *igm* can also be modulated in mucosal surfaces by abiotic stressors such as air exposure, as previously described in gills and intestine of stressed *Epinephelus coioides* and *Oreochromis niloticus* (72, 73). It has also been reported that environmental changes and also *V. anguillarum* increased the expression of pro- and anti-inflammatory cytokines in the gastrointestinal tract (70, 74, 75) and also in head kidney, spleen, and liver (76–78). It is worth mentioning that regulation of *igm* appears to be repressed by *A. salmonicida* in Atlantic cod (79), indicating a different regulation of *igm* when fish are exposed to an antigen compared to a pathogen exposure. Our findings confirm the relevance of duration and type of the stressors that affect particularly seabream mucosal tissues and suggest more pronounced effects of air exposure in seabream intestine. Altogether, the results suggest that in trout gut a modulation of particular genes will be activated depending on the type of stressor, in this case biotic or abiotic. On the other hand, the expression of pro/anti-inflammatory (*il1β, il6, tnfα, il10*, and *tgfβ1*) cytokines in seabream gut was mainly induced by air exposure, reinforcing the relevance of the abiotic stressor effect on seabream mucosal tissues. Thus, the induction of stress and immune genes expression was coincident with high levels of plasmatic and mucus cortisol. Overall, from our findings, intestine appears to be one of the most affected surfaces by different types of stressors, and in terms of gene expression, the gut mucosa shows higher sensitivity to air exposure than to vaccine.

### Overview of MALT Responses

Skin mucus cortisol level showed variations between species, and a clear difference was also observed in terms of stress- and immune-related gene expression. Skin and intestine appear to be the most affected surfaces after different types of stressors both in trout and seabream. When applying both stressors, skin particularly appears to be the most reactive barrier to vaccine + air exposure.

The extent to which husbandry conditions modulate mucosal immune response need to be much more investigated because of the complexity of the immune system and the interactive nature of the stress response. Thus, dealing with stressors of different features it may be problematic to predict the direction and magnitude of the response. Our results show, in general, an increased MALT response after the combination of stressors in seabream but not clearly in trout. Hence, previous studies showed higher response of innate indicators in low density than high density after bacterial exposure (80). One of the reasons that may explain why a combined stressor does not induce higher responses could be associated with the energetic load that concurrent challenges would require for such an increased response. Thus, the available energy would not be enough to meet the energetic needs.

Several reasons can be claimed to be responsible for the interspecific differences observed: one is the diversity of the species living in either marine or freshwater habitats. In fact, the differentiation of the fish population is eight times higher in freshwater than in seawater environments, which would support the differences among genomic architectures (81). Hence, the ecological characteristics of *V. anguillarum*, halophilic bacteria, may partially explain the observed differences. Outbreaks of *V. anguillarum* bacteria affect mainly marine and estuarine fish species at different salinities (usually 1–2% NaCl) and temperatures exceeding 15°C (82). *V. anguillarum* can also be found occasionally in freshwater, forming biofilms to enhance bacterial survival in an otherwise suboptimal environment (83). Therefore, freshwater fish (trout) would be more susceptible to *V. anguillarum*. Given that temperature seems to be more detrimental than salinity for *V. anguillarum* growth (7), our results suggest that cold freshwater trouts may not experience significant exposure to *V. anguillarum* in the natural environment, thus lacking an evolutionary-driven, parasite-tuned host–pathogen immune crosstalk. Therefore, the increased responsiveness to *V. anguillarum* bacterin observed mainly in trout may account in part for the upregulation of several key inflammatory transcripts that are downregulated in a marine fish such as seabream. A second reason would be related to the interaction of *V. anguillarum* with the fish microbiota, as freshwater or seawater fish can display rather different microbiomes. Thus, a data set analysis from a large collection of 16 S rRNA of diverse free-living and host-associated bacterial communities from intestines of different fish species suggests that variation in gut microbiota composition in fish is strongly correlated with species habitat, salinity, and trophic level (84). A third reason would be related to the salinity or temperature themselves. Thus, it has been shown that hyperosmotic and also hypoosmotic stress modify the immune homeostasis in catfish (85). However, in these experiments, fish were subjected to changes from their acclimated conditions, whereas in the present study, both species were well acclimated to their termopreferendum and natural salinity levels to precisely avoid potential stress biasing the data analysis.

Altogether, the interspecific differences in the regulatory responses observed under the different stressors suggest an adaptive lifetime in either freshwater or marine habitats resulting from a complex interaction between environmental conditions, microbial communities, and genomic variation that may affect the intensity and dynamics of the inflammatory and stress responses.

## Conclusion

Our findings illustrate the implication and importance of the mucosal immunity in response to different stressors and provide comparative data on the transcriptomic responses of several immunomodulators in MALT tissues. In species such as trout and seabream acclimated to their adaptive thermoneutral environments and confronted to *V. anguillarum* bacterin, our results show a higher responsiveness of skin and gills immune transcripts to the biotic stressor in trout than in seabream. On the other hand, in all mucosal organs evaluated, a higher response to the abiotic stressor was observed. Our results indicate that the response of the immune system is not homogeneous among mucosae and that is greatly influenced by the type of stressor, suggesting a trade-off between suppression and enhancement of immune responses depending on the intensity and duration of the stressors in each surface. In agreement with previous report in mammals and recent reports under *in vitro* conditions, our results clearly indicate distinctive responses of rainbow trout and seabream (86, 87). Considering the greater immune-related gene expression of seabream after stress in skin and gut, it can be suggested that mucosal tissues of gilthead seabream (a marine fish) show more responsiveness than rainbow trout (a freshwater species). This differential immune response can be attributed to the species specificity of the response, genetic diversity, or environmental conditions such as type and abundance of pathogens. This microorganism diversity may undoubtedly participate in explaining the different immune responses between fish, together with the microbiota, high or low salinity or higher or lower temperatures. However, the scarcity of studies on these environmental influences does not allow us to propose a consistent interpretation of those differences. Like mammals, the impact of acute stress and the consequent immunoendocrine reaction appears to enhance or modulate rather than always suppress the response of mucosal tissues. Thus, features of the stressors (type, intensity, and duration) determine the direction of the effect on mucosal immune system. Overall, and regarding the species differences, although our hypothesis is confirmed in the sense that the response to stressors is species-specific, we also show that such specificity is more intense, since two different species such as trout and seabream show not only quantitative but also qualitative differences in their responsiveness.

## Ethics Statement

The experiment complied with the Guiding Principles for Biomedical Research Involving Animals (EU2010/63), the guidelines of the Spanish laws (law 32/2007 and RD 53/2013), and it was authorized by the Ethical Committee of the Universitat Autònoma de Barcelona (Spain) for the use of laboratory animals.

## Author Contributions

AK performed the sampling, gene expression, analysis and interpretation of results, and wrote the manuscript. JB performed graphic presentation of results, manuscript design, interpretation of results, and wrote the manuscript. DP performed sampling and interpretation of results. EVV performed gene expression analysis and interpretation of results. FERL and LT conceived the study design, supervised the experiment, performed the analysis and interpretation of results, and wrote the manuscript. All authors read, corrected and approved the final manuscript.

## Conflict of Interest Statement

The authors declare that the research was conducted in the absence of any commercial or financial relationships that could be construed as a potential conflict of interest. The reviewer PC and handling Editor declared their shared affiliation.
